# A global coastal permeability dataset (CoPerm 1.0)

**DOI:** 10.1038/s41597-024-03749-4

**Published:** 2024-08-17

**Authors:** Nils Moosdorf, Jarrid Tschaikowski, Daniel Kretschmer, Robert Reinecke

**Affiliations:** 1 Leibniz Centre for Tropical Marine Research (ZMT), Bremen, Germany; 2 Kiel University, Kiel, Germany; 3 Johannes Gutenberg-University Mainz, Mainz, Germany; 4 Potsdam University, Potsdam, Germany

**Keywords:** Hydrology, Environmental sciences

## Abstract

The permeability of aquifers strongly influences groundwater flow characteristics. Worldwide, coastal groundwater is often the primary freshwater source for coastal communities and ecosystems but is also particularly vulnerable to abstraction since saltwater intrusion may threaten its quality. Thus, understanding coastal permeability is crucial to the sustainable use of coastal groundwater. Here, we present the first global dataset of coastal permeability (CoPerm 1.0), which provides data on coasts’ landward, shoreline, and seaward permeability. CoPerm accounts for shoreline characteristics such as cliffs and beaches and contains information on four million segments representing more than two million kilometers of global coastline. Rocky Shores are the most abundant shoreline class, followed by mangroves, beaches, and muddy coasts. Permeability differs between the immediate shoreline (median permeability: 10^−12.3^ m^2^), the seaward (median: 10^−13.3^ m^2^), and the landward (median: 10^−13^ m^2^) sides of the coast. CoPerm provides input data for global coastal groundwater assessments and regional studies of submarine groundwater discharge or saltwater intrusion that can radiate into ecological and economic studies.

## Background & Summary

Groundwater is a crucial freshwater resource for humans and ecosystems that is under intensive pressure worldwide^1–3^. Groundwater at the coasts is particularly vulnerable to abstraction and climate change^4–6^. The threat of seawater intrusion to groundwater quality is amplified by sea level rise^7,8^, while pumping of coastal groundwater can lead to land subsidence and flooding^9^. Since population density at coasts is very high, groundwater conservation is particularly urgent there, as it often is the only source of available freshwater^10^.

In addition to its relevance as a source of fresh water, fresh submarine groundwater discharge is a transport pathway for nutrients^11,12^ and trace elements^13^. The nutrient fluxes have been demonstrated to affect coastal ecosystems worldwide^14–16^. Groundwater poses a risk of eutrophication in 25% of the estuarine coasts worldwide^12^, entailing that coastal groundwater deserves particular attention at a global scale.

Permeability, the capacity of rocks to transmit fluids, is essential to subsurface groundwater flows. It determines the flow rates for given hydraulic gradients and aquifer geometry. At the global scale, the permeability of aquifers was estimated based on global lithological maps^17–19^. Yet the lithological maps that are the foundation of global permeability maps, e.g.^20^, were focused on land areas and did not pay particular attention to the coastline. Notably, the coasts are linear features with high variability that require a different approach than inshore permeability. While it is even difficult to define the exact position or length of the coastline^21^, the linear coastal features have a tiny areal footprint. Still, they can strongly influence groundwater-ocean connectivity^22,23^. The existence of coastal features such as beaches^24^, reef plates^25^ or burrows in fine-grained sea-bottom sediment^26^ can be relevant to coastal groundwater flows and biogeochemistry but are usually not represented by the available regional terrestrial aquifer information.

**Fig. 1:**
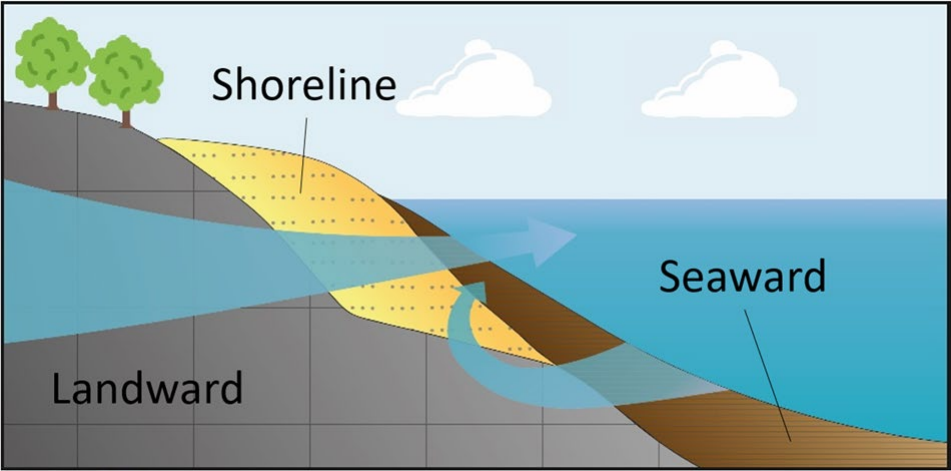
Three aspects of coastal permeability. This study considers the landward aquifer, the immediate shoreline, and the seaward coastal marine bottom sediment. The figure is simplified and just aims at illuminating the nomenclature used in the dataset.

Introducing the coast into earth system models based on synthesizing different coastal attributes will be important for our understanding of the earth system and is one of the grand challenges of that field^27^. While attempts have been made to characterize regional coastal aquifers^22,28^, the specifics of the immediate coastline have yet to be considered globally. Here, we present a dataset integrating interdisciplinary input data to address three different permeability values for the global coasts, onshore aquifers, the immediate coastline, and offshore coastal sediment (Fig. 1). The immediate coastline is here defined as the few 100 s of meters around the coast. A lot of it usually would consist of the intertidal area, where the most intensive groundwater ocean interaction takes place^29^. The seafloor is thought to represent subtidal areas down to a few tens of meters water depth. However, there is no clearly defined boundary, given also the nature of the data. The dataset can provide a basis for large-scale coastal groundwater studies and representation of the coast in earth system models.

**Fig. 2:**
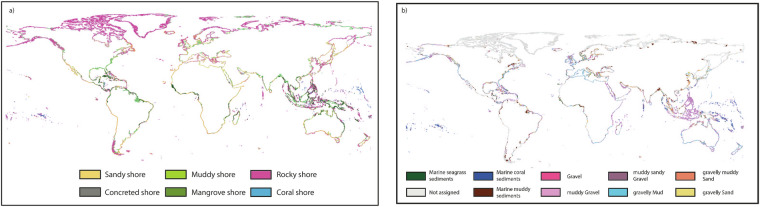
Global distribution of (**a**) shoreline permeability and classes and (**b**) seaward permeability classes.

**Fig. 3:**
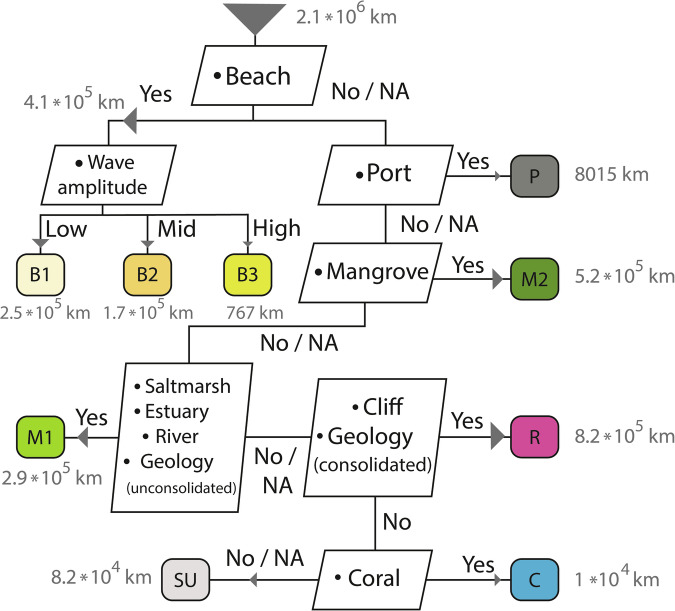
Decision tree for the shoreline classification into different permeability classes and their assigned shoreline length (in grey; total combined shoreline length: 2.1 * 10^6^ km). Class names: B1: Sandy shore low waves; B2: Sandy shore mid waves; B3: Sandy shore high waves; P: concreted shore; M2: Mangrove shore; M1: Muddy shore; R: Rocky shore; C: Coral shore; SU: Not assigned shoreline.

**Fig. 4:**
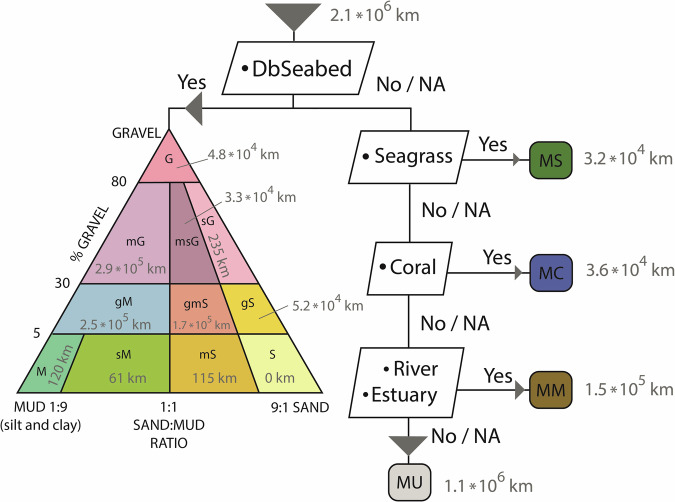
Decision tree for the seaward permeability classification into the different permeability classes and their assigned shoreline length (in grey; total combined shoreline length: 2.1 * 10^6^ km). MS: Marine seagrass sediments; MC: Marine coral sediments; MM: Marine muddy sediments; MU: Not assigned for seaward view; G: Gravel; mg: muddy Gravel; msG: muddy sandy Gravel; sG: sandy Gravel; gM: gravelly Mud; gmS: gravelly muddy Sand; gS: gravelly Sand; M: Mud; sM: sandy Mud; mS: muddy Sand; S: Sand.

The landward, shoreline and seaward permeabilities could be classified along more than 90% of the global coastline (Table 1), whereas a seaward permeability could be assigned for about half of the coast. The unassigned values for seaward permeability are primarily situated in the far north, where few DbSeabed data are available (Fig. 2). Landward permeability was taken directly from the GLHYMPS data^18,19^. The most abundant shoreline permeability class was Rocky Shores (Table 1, Fig. 3). While this aligns with previous work reporting a dominance of rocky shores along the global coastline^30^, their frequent occurrence along the northern coasts could also partly be attributed missing data which would have led to other classifications, paired with an emphasis on bedrock in the lithological source data (see decision tree in Fig. 4). Sandy coasts are found, e.g., around the western coasts of the Americas, and muddy shores are assigned to large parts of central Europe and most of the Brazilian coast (Fig. 2). The most abundant seaward permeability was muddy gravel (Table 1, Fig. 4). The seaward permeabilities were equally distributed between the dominating classes over the entire coastline (Fig. 2).

**Table 1:** Describing statistics of the landward, shoreline and seaward permeability values of the global coast.

	Landward	Shoreline	Seaward
Length	2.1 * 10^6^ km (total coast length)		
Percent classified	92.9%	96.2%	49.7%
Most abundant coastal class (and its length)	n.a.	Rocky Shores (8.2*10^5^ km)	Muddy Gravel (2.9 * 10^5^ km)
Median permeability	10^−13^ m^2^	10^−12.3^ m^2^	10^−13.3^ m^2^

**Fig. 5:**
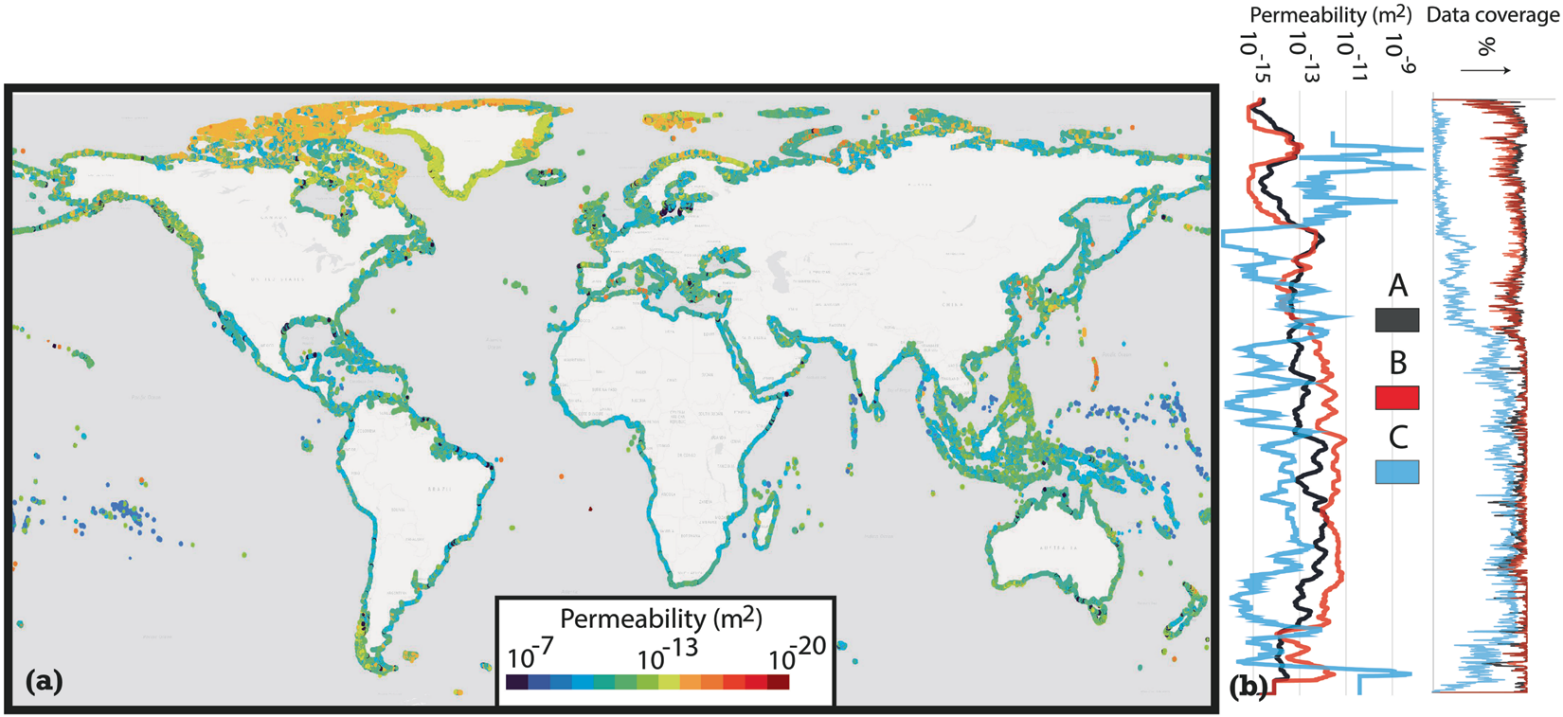
Global distribution of the coastal permeability. The figure shows the median of the landward, shoreline, and seaward permeabilities.

The translation into permeability shows that the median landward permeability is 10^−13^ m^2^, the median shoreline permeability is 10^−12.3^ m^2^, and the median seaward permeability is 10^−13.3^ m^2^ (Table 1). Shoreline permeability is generally the highest, while seaward permeability is the lowest, particularly towards the equator (Fig. 5). In contrast, in the northern and southern latitudes, seaward permeability is higher than the others (but with a low data coverage).

This study is the first global permeability dataset focusing on the ocean coast, but its collection of coastal attributes can also be used for other applications. While the uncertainties of the dataset are substantial, they are very hard to quantify. Geographically, the coastline features and many input data have a very high spatial resolution of up to 30 m. However, thematic uncertainty remains very high. Classes could have been categorized into the wrong class, based on wrong data inputs (e.g. errors in land cover dataset classification) or unavailable data. Then, the classification system can yield unlikely results (as e.g. for the rocky coasts in large parts of northern Asia). In some cases, the classification itself can be misleading, e.g. in the case of sandy cliffs. To keep the decisions transparent and the data replicable, the decision tree is clearly explained and no exceptions were made.

The here applied approach to sort the coastline into classes and assign one permeability to each specific class implies the permeabilities of each coastline class were the same across the globe. This substantial simplification is necessary to produce a reasonable value, but the translation between coastal features and permeability adds uncertainty, since permeability of individual sediment types, particularly on the seafloor, can vary substantially, based on local conditions^17,31^. The attributes of beaches, for example, can be influenced by the provenance of their sediments, as shown e.g. in Mallorca^32^ and Victoria (AUS)^33^. Their shape and form is controlled by their morphodynamic positioning at the coast^34^. Sediment permeability also changes strongly with depth of the sediment^35^ and based on sediment layering, permeability can differ in horizontal and vertical direction. Locally, bioturbation, such as crab burrows, can significantly alter permeability^36,37^. This, and effects of storm reworking of sediment^38^, means that coastal permeability does not just change over space and depth, but also over time. The values for mud that are used here are lower than they may be at the immediate surface and also do not account for effects of macropores, which may locally be significant^39^. Such grade of detail can at this time not be represented at the global scale based on existing data. Quantifying permeability at the global scale is a compromise and will not be accurate for the local scale. Thus, the values provided here are highly uncertain and should not be applied for local scale problems of individual locations. A likely range of expectable permeability values can serve as estimate for uncertainty (Table 2). Yet, the uncertainty ranges provided in this study are taken from literature values and can seem narrow, e.g. for sandy shores. The values were selected to be broadly usable, however, for each individual application of the dataset for any specific problem, it is worth evaluating if the provided values are usable, or if they need to be adapted. Adaptation is possible using the provided code^40^. The seaward permeability still has a lot of unknown areas, particularly where DBSeabed data were missing, and the places where it is based on DBSeabed data are influenced by a certain overestimation of the gravel content and an underestimation of the unmixed sand classes because of interpolation mechanics between the DBSeabed points (personal communication by Chris Jenkins, the author of the dataset).

**Table 2:** Assigned permeability values and ranges for the different permeability shoreline classes with an explanation.

Permeability Class	Permeability value (m²)	Permeability Range (m²)	Permeability source
**Sandy shore (B1 - Low Waves)**	10^−11^	10−^11^–3*10−^11^	Value: Lowest value of beach the permeability range stated by^61–64^ Range: Whole range for all beach literature values stated
**Sandy shore (B2 - Mid waves)**	2*10^−11^	10−^11^–3*10^−11^	Value: Middle value of beach permeability range stated by^61–64^ Range: Whole range for all beach literature values stated
**Sandy shore (B3 - High Waves)**	3*10^−11^	10^−11^–3*10^−11^	Value: The highest value of beach permeability range stated by^61–64^ Range: Whole range for all beach literature values stated
**Concreted shore (P)**	10^−20^	10^−20^–10^−20^	Considered as Impermeable
**Muddy shore (M1)**	5*10^−13^	10^−13^–10^−12^	Value: Average of the range of literature values^65–69^
**Mangrove shore (M2)**	5*10^−12^	10^−12^–10^−11^	Value: Average of range^70,71^
**Rocky Shore (R)**	Permeability taken from^19^	The range was taken from^19^	Rocky shorelines are assigned the permeability values stated by^19^ for that location.
**Coral shore (C)**	1.2*10^−10^	2.9*10^−11^–4.6*10^−10^	Average of range for coral sand^72^

Yet, the presented dataset will prove helpful in global scale coastal groundwater modelling and geochemical flux estimates at the coast. It will be improved with the availability of new data and will enable large-scale coastal groundwater assessments and products applying their results.

## Methods

Permeability at the coastline was classified for the landward aquifer, the immediate shoreline, and the coastal seafloor sediment at the seaward side of the coast. The physical shape of the coastline is defined by a 30-meter resolution global shoreline vector database^41^ available for download. The global coast (which excludes Antarctica) has a total length of approx. 2.1 million km and was cut into 4.005 million pieces of ≤1 km (in the following termed “coastline vector”). This dataset already provides a set of environmental information^42^, which was complemented with additional data for this study.

Unless explicitly stated, spatial procedures were done using the software QGIS 3.22.4. Depending on the parameter dataset (vector type, raster resolution), either the original line segments or midpoints of the line segments (provided in the initial global shoreline vector database) were used for the parameter value assignment to the coastline vectors.

All input data are publicly available for download where mentioned in the references. The code used to derive the dataset and described in plain words in the methods section is available for download from Zenodo^40^.

The data of the CoPerm v1.0 are available for download from PANGAEA^43^.

### Landward permeability

The landward permeability represents the coastal aquifer that extends on land. It can be relevant for regional scale saltwater intrusion studies e.g.^7^, as well as studies of coastal groundwater availability e.g.^10^. This part of coastal permeability is already represented best by global permeability datasets, namely GLHYMPS^18,19^. Thus, the landward permeability for our database was derived from the permeability dataset GLHYPMS 2.0^19^, which represents the regional scale landward aquifer.

To extrapolate the permeability data to coastline segments slightly outside the GLHYPMS 2.0 coverage because of a different definition of coast, GLHYPMS 2.0 was rasterized with a cell size of 1 km using the permeability as raster value and the provided standard variation for uncertainty analysis and converted into a 16-bit signed integer type. To cover areas slightly outside the original data, the raster was extended by 5 km (5 grid cells) using the Focal Statistic tool in ArcGIS Pro with a neighborhood circular setting with a radius of 5 and statistic type ‘mean’. The GLHYPMS 2.0 original and extended raster were then merged with the GDAL tool ‘Merge’, favoring the original raster where it had data. This produces an output of the GLHYPMS raster extended by 5 km with the average permeability of the neighboring cells. The spatially extended permeability raster was joined to the coastline segment midpoints using the QGIS ‘Pointsampling’ tool. By spatially joining the point sampling results to the coastline segment midpoints (using the QGIS tool join by location), the coastline segments received their permeability information.

### Shoreline permeability

The immediate coastline is a highly reactive zone that controls chemical fluxes and interaction between groundwater and the ocean^44^. It is essential for studies assessing the biogeochemical role of the subterranean estuary e.g.^45,46^. The existence of a beach, for instance, can change water flows strongly. To define a permeability classification, several datasets providing information about attributes of the immediate coastline were combined. Based on these attributes, the coastline was divided into permeability-related classes in a defined decision tree (Fig. 3). The order of the decision tree is based on the impact of the attribute on permeability, its thematic specificity, and the spatial resolution of its input data. The description of the individual input data in this chapter follows the order shown in the decision tree (Fig. 3).

The existence of beaches was derived from a global beach occurrence dataset^47^. It identified sandy beaches every 500 m of the coast by applying a pixel-based supervised classification to a high-resolution global composite satellite image of 2016. The point data set is binary, with 1 for Sandy Beach and 0 for no beach. The beach occurrence data and the coastline segment midpoints were buffered by 0.5 km to merge the point data information to the coastline vector. That ensures an overlay of the buffers as the spacing of the beaches is denser than that of the coastline segment midpoints of 1 km. Both buffers were then intersected, and the result dissolved with the GDAL tool per coastline segment, the maximum identification value per segment. This ensures that each coastal segment within a beach occurrence within a radius of 1 km is assigned a beach value.

An approximate grain size was estimated for beaches based on wave amplitude at those sites in three broad categories (B1, B2, B3), for low, medium and high wave energy. Wave amplitude per coastal segment was provided in the original shoreline data^42^.

To represent artificial coasts (class P), point data of global ports of the World Food Program^48^ were used. The points represent locations of 3581 Ports sorted into size classes. To integrate artificial/concrete coastlines along the ports, examples of the different port sizes were first reviewed in Google Maps and assigned an affected length of the coast (Big = 5 km; Large = 3 km; Medium = 2 km; Small = 1 km; Very Small = 0,5 km; Unknown was set as the average of all class sizes to 2.3 km). The port points were then buffered in QGIS with a radius according to their assigned port size. The buffers were intersected with the coastline segment midpoints and dissolved to avoid duplications.

One way to learn about the characteristics of coastal sediments is by covering coastal ecosystems. Therefore, the World Atlas of Mangroves (v. 3.1)^49^ was employed to define coastal segments as a mangrove class (M2). Mangroves tend to indicate a muddy-sandy coastline. The global mangrove distribution coastlines’ 30-meter resolution polygon vector data are similar to the coastline segments. For the merging process, the coastline segment midpoints were buffered with a width of 1 km. The Buffer was then intersected with the mangrove polygon vector data. The resulting intersection was then dissolved by coastal segment to avoid duplications. The mangrove dataset is high in the decision tree because of its high spatial resolution and specific nature.

Saltmarshes, estuaries, rivers, and unconsolidated geology define the mud-influenced shoreline class (M1), which is here called “muddy” for simplicity. We used the global saltmarsh map (v6.1)^50^ to classify coastal segments as saltmarshes. The dataset contains vector polygons of saltmarsh distribution in 99 countries with raw data of scales from 1:10.000 to 1:4.000.000, most finer than 1:100.000. Since the saltmarsh coastlines aligned well with the coastline segments, the datasets were spatially superimposed with a snap geometry to layer tool. For this, the coastline segment midpoints were chosen as the input layer, and the salt marsh vector was chosen as the reference layer with a tolerance of 500 m. The snapped midpoints were joined to intersecting salt marsh polygons by location.

The shorelines of coastal segments were classified as estuaries if they were within 2 km of the global estuary database V 2.0^51^. All distances mentioned here are based on assumptions on the effect scale and the resolution of the input data and represent a compromise between the goal to provide as many values as possible and the uncertainty increasing with distance from the original data. The estuary database is a polygon shapefile containing 1201 estuaries. The estuary polygons were joined to the coastline segment midpoints by buffering those with a radius of 2 km. These buffers were intersected with the estuary polygons, and the intersection was then merged by segment (QGIS tool: ‘dissolve’) to avoid duplications.

The coastline segments follow the coast inland along large rivers at many locations. Our database represents this by classifying shorelines of coastal segments closer than 500 m to a river in the global large river dataset^52^ as ‘river’. For this, the polyline river data set was first buffered with a width of 10 meters. Then, the coastline segment midpoints were buffered with a radius of 500 m. Midpoint buffers were then intersected with river polygons, and the results dissolved to avoid duplications.

Another indicator for mud-influenced coasts is unconsolidated geology at places where no beach is indicated in the dataset. Unconsolidated geology is represented by the global lithological map GLiM^20^. The QGIS tool ‘Snap geometries to layer’ was used to spatially superimpose coastline segment midpoints on the closest lithology polygon. Since the coastline of the GLiM dataset has a coarse resolution, we ran the procedure two times, first with a snapping distance of 5 km, and increased the tolerance to 10 km in the second run. Both snapped coastline segment midpoints were spatially joined with the GLiM polygons. The resulting points were then spatially merged with the lithology data using the join attribute by location tool in QGIS. In R Studio, the merged coastline segment lithology data with the tolerance of 5 km and 10 km were then merged, preferring data of the 5 km tolerance run.

The class rocky shore (R) is based on three methods. First, they are classified when the sloping data in the original coastline vectors^42^ reported the segment as “steeply sloping” or “vertical.” Second, they were classified using a global cliff probability dataset^53^. The cliff data were points of cliff probability in percent, spaced approximately every 800 meters alongshore. The percentage was interpolated along the shoreline using the ArcMap 10.5 IDW tool (output cell size 1 km, power 2, up to 3 input points within a distance of 1000 m). The interpolated cliff probability value was extracted to the coastline segment midpoints using the ‘Extract Values to Points’ tool in ArcMap 10.5. Cliff probabilities of at least 50% were classified as rocky shore. Lastly, coastal segments with consolidated GLiM lithology and without indicators for mud-influenced coasts or beaches are classified as rocky shores.

Since no information on coastline types is available for many small islands, many of which are carbonate islands, the particular coral coast class (C) was added based on the global distribution of warm-water coral reef data (v. 4.1)^54^ for coastal segments closer than 1 km to a coral reef. The coral reef data were polygons with a resolution of 30 m and derived using high-resolution satellite imagery. To transfer coral reef occurrence to the coastline segments, the coastline segment midpoints were buffered with a width of 1 km and then intersected with the coral polygon vector data. The result was dissolved per segment to avoid duplicates.

All remaining coastal segments are classified into unknown information (SU).

After the classification of the coastal segments, each class was assigned permeability values based on ranges of permeability values identified in the literature (Table 2).

### Seaward permeability

The third aspect of coastal permeability is that of the marine sediment on the coast. This can be important when addressing porewater flow and associated solute flux^55^ but also for studies interested in coastal stability. The seaward permeability was determined based on sediment information derived from the DBSeabed database (developed and supplied by Chris Jenkins, INSTAAR). Where no data from DBSeabed were available, the classification relied on other datasets in a decision tree similar to the one presented for the immediate shoreline above (Fig. 4).

The DBSeabed database creates unified, detailed mappings of the seafloor material by integrating thousands of individual datasets, mostly from drill logs. We used a point data output from the database where a Compositional Data Analysis was applied to gravel:sand:mud (g:s:m) data to treat the closed triplet structure of the data. To perform statistical, graphical, and geometrical (including gridding) operations, the g:s:m data were transformed into three log ratios of the geometric mean (“centered log ratios”).

DBSeabed data points with a maximum water depth of 40 meters were extracted in R-Studio individually for centered log ratios of gravel, sand, and mud. The point data were separately interpolated into raster files in ArcMap 10.5 using inverse distance weighting (IDW; cell size of 1 km, search distance of 20 km, 12 points, power of 2). The interpolated raster values for the corresponding coastline segments were then sampled with the ‘Point sampling’ tool in QGIS and joined to the coastline segment features by location. Rasters of gravel/sand/mud proportion on the seabed sediments were retrieved using exp(CLR_sand, gravel, mud_)/Σ(exp(_sand, gravel, mud_))*100 and linked to coastal segments, as previously described for raster data. Based on the gravel proportion and the mud/sand ratio, the coastline segments were sorted into sediment classes following the system set up by Folk^56^ (Table 3).

**Table 3:** Sorting conditions of DBSeabed gravel, sand, mud percentage into Folk classification (compare Fig. 4 for a visual representation of the classification system).

Folk class	Defining condition
Gravel (G)	Gravel % ≥ 80%
muddy Gravel (mG)	80% < Gravel % ≥ 30% & Muddy % ≥ Sand %
gravelly Sand (gS)	30% < Gravel % ≥ 5% & Sand % > Sand % & Mud % * 9 < Sand %
Mud (M)	Gravel % < 5% & & Mud % > Sand % * 9
Sandy Mud (sM)	Gravel % < 5% & Mud % > Sand % & Sand % * 9 ≥ Mud %
muddy sandy Gravel (msG)	80% < Gravel % ≥ 30% & Sand % > Sand % & Mud % * 9 ≥ Sand %
muddy Sand (mS)	Gravel % < 5% & Sand % > Mud % & Mud % * 9 ≥ Sand %
sandy Gravel (sG)	80% < Gravel % ≥ 30% & Sand % > Sand % & Mud % * 9 < Sand %
gravelly Mud (gM)	30% < Gravel % ≥ 5% & Muddy % ≥ Sand %
gravelly sandy Mud (gmS)	30% < Gravel % ≥ 5% & Sand % > Sand % & Mud % * 9 ≥ Sand %
Sand (S)	Gravel % < 5% & Sand % > Mud % * 9

The Folk-Classes were adopted here as coastline permeability classes.

At coastlines outside a 20 km radius of DBSeabed-points, the seaward permeability was determined using a classification based on seagrass (class MS), coral (class MC), estuary, and river datasets (class MM) (Fig. 3). For classifying coastline segments into the seagrass class, we used a dataset of the global distribution of seagrasses (version 7.1)^57^. The vector polygon dataset contains reviewed seagrass locations of 128 countries and territories. Merging to the coastline segments was performed by buffering the coastline segment midpoints with a radius of 1 km, intersecting the midpoint buffers with the seagrass polygons, and dissolving the resulting intersection to avoid duplications. Treatment of the coral, estuary, and river datasets was described above. However, for the seaward classification, a longer radius of buffering was used for estuary (5 km) and river (2 km) influence, representing an assumed broader impact of those structures on the seaward permeability, as inferred from river sediment plume shapes^58,59^. The remaining coastline is in the class MU, indicating an unknown seaward permeability.

Each class was assigned a seaward permeability based on literature values (Table 4). While for the coastal classes based on the Folk classification system, the permeability for those respective grain sizes was sought in the literature, the ecological classes are defined based on the ecosystem needs. Seagrass tends to live on fine-grained sandy-muddy sediments, while the mud can’t dominate, so the grass still gets enough light through the water column. Similarly, corals usually live on hard substrate or coral rubble but do not tolerate high water turbidity and high mud content. On the contrary, river and estuary seaward sediments are expected to have a high mud content and are classified as such.

**Table 4:** Assigned permeability values and ranges for the different permeability seaward view (shallow marine sediments) classes and folk classification with an explanation.

Permeability Class	Permeability value (m²)	Log *100 Permeability	Permeability Range (m²)	Log Permeability * 100 Range	Comment
Gravel (G)	5*10^−8^	−730	10^−10^ – 10^−7^	−700 – (^−^1000)	Value: Average of range for gravel permeability^73^.
muddy Gravel (mG)	5*10^−14^	−1330	10^−16^ – 10^−13^	−1300 – (−1600)	Value: Average of range for muddy gravel^74^.
muddy sandy Gravel (msG)	3.9*10^−11^	−1041	5*10^−14^ – 5*10^−8^	−730 – (−1330)	Value: Geometric mean of G, sG, mG & gmS permeability values. Range: Range of the direct neighbor field values.
sandy Gravel (sG)	3.4*10^−10^	−947	3.9*10^−11^ – 5 * 10^−8^	−730 – (−1041)	Based on grain size analysis^75^ – assumed composition: d60 = Gravel (2 mm), d10 = Sand (0,63 mm) = U: 3.17. Range: Range of direct neighboring fields.
gravelly Mud (gM)	5*10^−17^	−1630	10^−19^ – 2.7 *10^−12^	−1157 – (−1900)	Value: Average of permeability range of unweathered marine clay^73^. Range of the direct neighbor fields. Range: Range of direct neighbor fields.
gravelly sandy Mud (gmS)	2.7*10^−12^	−1157	10^−17^ – 1.8*10^−10^	−976 – (−1630)	Grain size analysis^75^ – assumed composition: d60 = Sand (0.6 mm), d10 = Mud (0,06 mm) = U: 10. Range: Range of the direct neighboring fields.
gravelly Sand (gS)	1.8*10^−10^	−976	2.7 * 10^−12^ – 3.4 * 10^−10^	−947 – (−1157)	Value: Geometric mean of sG, S & gmS. Range: Range of direct neighbor fields.
Mud (M)	10^−19^	−1900	10^−19^ – 10^−16^	−1600 – (−1900)	Value^76^: and lower Range stated by^73^. Range^73^:
Sandy Mud (sM)	5*10^−17^	−1630	10^−19^ – 10^−14^	−1400 – (−1900)	Value: Average of permeability range of unweathered marine clay^73^. Range of the direct neighboring fields.
muddy Sand (mS)	1.7*10^−14^	−1376	5.5 * 10^−16^ – 5.5 *10^−13^	−1226 – (−1526)	Average of the range stated by^74,77^ value stated by^76^->20% Clay, Fig. 4.
Sand (S)	10^−11^	−1100	10^−13^ – 10^−9^	−900 – (−1300)	Average of range for clean sand permeability^73^.
Marine seagrass sediments (MS)	1.7*10^−14^	−1376	5.5 * 10^−16^ – 10^−11^	−1100 – (−1526)	Most seagrass species grow on sandy to muddy sediments^78^. Seagrass can’t grow if the substrate is too muddy^79^. MS permeability is chosen to be like Folks class muddy Sand (mS) – Range from clean sand to lower end of muddy Sand.
Marine coral sediments (MC)	1.2*10^−10^	−992	2.9 * 10^−11^ – 4.6 * 10^−10^	−933 – (−1053)	Value: Average of range for coral sand^72^.
Marine muddy sediments (MM)	5*10^−17^	−1630	10^−19^ – 10^−14^	−1400 – (−1900)	Value: Average of Range. Range: Range for marine clay^73^.

Some of the comments refer to the Folk-Diagram shown in Fig. 4.

## Data Records

The here-described data records on PANGAEA (https://doi.pangaea.de/10.1594/PANGAEA.958901)^43^ encompass a technical description (Filename: CoPerm_Technical_Appendix.v.1.0.pdf) and a zip archive (filename: CoPerm_.v.1.0_Data.zip) containing the actual data in CSV format (filename: CoPerm_v.1.0_Dataset.csv) and a set of metadata (filename: CoPerm_v.1.0_Metadata_Descriptor.txt). In that metadata set, all columns in the data table are described.

Geographical features are not included in our published data to avoid double publication of data. The table has to be joined to the original geographical features of the coastline that are easiest available from a derivate produced from the original author at 10.5066/P9HWHSPU^60^ (File: USGSESRIGlobalCoastalSegmentsv1.mpk) using the column “MasterKey” in both datasets. The coastal permeability values presented here are fully usable only joined to those data.

## Technical Validation

Due to the global nature of the dataset and the available observational data at this scale, we do not see a formal way to validate the global coastal permeability data. We thus chose to be as transparent as possible in our decision-making, based on published literature throughout the process, from classification to assigning a permeability class. The dataset is built so that even if users disagree with any individual decision or permeability attribution, they can easily change it and insert their preferred values. Given the published literature values of permeability, the dataset is as robust as possible. To represent its uncertainty, we added permeability ranges to the coastal segments. We have further checked and re-checked the relations between decisions, coastal positions, coastal categories, and permeabilities. We have internally reviewed the code and all datasets manually. The decision trees are documented above; the code is published (see below).

## Data Availability

The code developed to derive classification decisions and permeability assignments, as described before, is available at: 10.5281/ZENODO.7845568^40^.
